# Body composition of the upper limb associated with hypertension, hypercholesterolemia, and diabetes

**DOI:** 10.3389/fendo.2022.985031

**Published:** 2022-08-31

**Authors:** Qianjin Qi, Kui Sun, Ying Rong, Zhaoping Li, Yixia Wu, Di Zhang, Shuaihua Song, Haoran Wang, Li Feng

**Affiliations:** ^1^ Department of Clinical Nutrition, Shandong Provincial Hospital Affiliated to Shandong First Medical University, Jinan, China; ^2^ Department of Radiology, Shandong Provincial Hospital Affiliated to Shandong First Medical University, Jinan, China; ^3^ Shandong Provincial Hospital Affiliated to Shandong First Medical University, Jinan, China; ^4^ Shandong Provincial Hospital, Cheeloo College of Medicine, Shandong University, Jinan, China

**Keywords:** body composition, obesity, hypertension, hypercholesterolemia, diabetes

## Abstract

The associations between segmental body composition and metabolic diseases remain equivocal. This study aimed to investigate this association using the example of U.S. adults. This cross-sectional study included 12,148 participants from the National Health and Nutrition Examination Survey (NHANES) (2011-2018). Multivariable logistic regression models were used to estimate associations between segmental body composition quartiles of hypertension, hypercholesterolemia, and diabetes. Among 12,148 participants, 3,569, 5,683, and 1,212 had hypertension, hypercholesterolemia, and diabetes, respectively. After adjusting for potential confounders, increased percent upper limb lean body mass was associated with a lower risk of hypertension (OR= 0.88, 95%CI: 0.84, 0.92, P _trend_<0.001), hypercholesterolemia (OR= 0.93, 95%CI: 0.89, 0.96, P _trend_<0.001), and diabetes (OR= 0.96, 95%CI: 0.95, 0.98, P _trend_<0.001). Increased upper limb fat mass is associated with an increased risk of hypertension (OR= 1.11, 95%CI: 1.07, 1.15, P _trend_<0.001), hypercholesterolemia (OR= 1.05, 95%CI: 1.01, 1.09, P _trend_=0.07), and diabetes (OR= 1.03, 95%CI: 1.01, 1.05, P _trend_=0.014). The same correlations were found in the torso and whole-body composition parameters. We observed that for women, lean body mass has a better protective effect on metabolic diseases [hypertension (OR= 0.88, 95%CI: 0.82, 0.93), hypercholesteremia (OR =0.86, 95%CI: 0.81, 0.92), diabetes (OR= 0.97, 95%CI: 0.85, 0.99)]; for men, increased body fat is associated with greater risk of metabolic disease[hypertension (OR= 1.24, 95%CI: 1.15, 1.33), hypercholesteremia (OR =1.09, 95%CI: 1.01, 1.18), diabetes (OR= 1.06, 95%CI: 1.01, 1.10)]. There were significant differences between different gender. These findings suggested that upper limb and torso adiposity should be considered when assessing chronic metabolic disease risk using body composition.

## Introduction

Metabolic disease (MD) consists of various metabolic abnormalities, including hypertension, hyperlipidemia, and diabetes (1). According to data released by the National Health and Nutrition Examination Survey (NHANES), the incidence of metabolic syndrome is 24% and 22%, respectively, in men and women (2). So, MD is an emerging and severe public health concern worldwide (3). Hypertension and pre-hypertension are responsible for 8.5 million deaths from stroke, ischemic heart disease, other vascular diseases, and renal disease worldwide (4). Hypercholesterolemia is generally accepted as the second most crucial risk factor for developing cardiovascular disease after hypertension (5) and is a modifiable factor (6). Diabetes has become the ninth leading cause of death, and more than one million people die each year of diabetes (7). The global population with diabetes is projected to be 700 million by 2045 (8). Recently, various studies investigated risk factors of MD, but the current understanding remains incomplete.

However, identifying potentially modifiable risk factors is vital in preventing and managing MD (9), and obesity is one of the modifiable factors. Numerous studies linked obesity with a higher risk of hypertension, hypercholesterolemia, diabetes, and death (10, 11). Previous NHANES study has shown that dyslipidemia is the most common co-morbidity related to obesity, followed by hypertension and diabetes (12). It may be due to hormone changes, inflammation, oxidative stress, and insulin resistance levels (13–15). Usually, we use Body Mass Index (BMI) to reflect obesity, but BMI cannot accurately reflect body composition. Recent studies have proposed the “obesity paradox” (16, 17). Furthermore, the relationship between BMI and MD may vary by race (18–20) and gender (21, 22). so knowledge of body composition will help better understand the relationship between obesity and obesity-related metabolic risks (23, 24).

The body composition assessment is one of the cornerstones of studying human metabolism and physiology (25). Segmental body composition parameters may better reflect the effects of obesity (26, 27) and have received much attention in recent years. These parameters can be quickly assessed using dual-energy X-ray Absorptiometry (DXA). Calculating the masses of different components using two X-ray attenuators and measuring segmental body composition by subdividing the body using specific, well-defined cut lines (28). DXA is the preferred method for body composition (28) and has been widely used (29–31). Body fat indices measured by DXA may help further identify people at risk for hypertension even when they have normal BMI (32).

The relationship between body composition and MD has been studied (33). However, few studies have been conducted on segmental body composition parameters and MD. The connections between segmental obesity and MD remain equivocal. For example, studies have found no strong evidence that body composition is a significant determinant of hypertension and diabetes (34, 35). In contrast, a study from the UK showed that hypertension was directly related to a fat mass percentage (FM%) and inversely associated with lean mass percentage (LM%) (36). Diabetes is associated with reduced LM%, but the relationship between FM% and diabetes is unclear (37). Besides, few studies on the relationship between hypercholesterolemia and body composition. Notably, total FM% or LM% may not reflect specific segmental obesity status. Therefore, we evaluated FM% and LM% of each body segment to clarify the relationship between segmental obesity and MD.

This study aimed to investigate the associations of segmental body composition with hypertension, hypercholesterolemia, and diabetes.

## Materials and methods

### Study population

NHANES is a multistage, nationally representative study designed to assess health and nutrition measurements (38). NHANES collected person-level demographic, health, and nutrition information from personal interviews and a standardized physical examination in a mobile examination center (MEC) (39). The survey examines a nationally representative sample of approximately 5,000 people every year. NHANES was performed by the National Center for Health Statistics (NCHS) of the Centers for Disease Control and Prevention (CDC) and was approved by the institutional review board of the National Center for Health Statistics. All participants signed a written informed consent form.

DXA is usually only performed in people aged 8-59. We restricted the analysis for this study to people aged 20 to 59 who were eligible for DXA examinations between 2011 and 2018. Pregnant women and people who weighed more than 450 pounds or were taller than 6’5” were already prohibited from DXA. Due to body components outside the scan region, alignment issues, overlapping arms or legs, excessive X-ray noise brought on by morbid obesity, and other factors that prevented the body area from being adequately evaluated, DXA results were considered invalid. Finally, 12148 participants were enrolled in the study.

### DXA measurements

DXA scan was performed using Hologic Discovery model A densitometers (Hologic, Inc., Bedford, Massachusetts), using software version Apex 3.2. Original scan results were analyzed with Hologic APEX version 4.0 software with NHANES BCA option to derive fat and lean mass. Trained and certified radiology technologists administered the DXA examinations. The University of California, San Francisco (UCSF) reviewed and analyzed each participant and phantom scan using standard radiologic techniques and NHANES-specific protocols. To ensure the accuracy and consistency of the results, the UCSF conducted expert reviews on all of the analyzed participant scans (40–43).

The torso region was defined as the area from the inferior edge of the chin as the upper borders to the oblique lines that cross the femoral necks and converge below the pubic symphysis as the lower perimeter, with vertical boundaries lateral to the ribs. The area below the lower borders of the torso was defined as the leg region (44, 45). Fat mass/lean mass was divided by segment weight to determine the segmental FM% and LM%. The left arm LM%, for instance, is calculated by dividing the left arm lean mass by the entire mass of the left arm.

### Main outcome

Hypertension was defined as systolic blood pressure (SBP) ≥ 140 mmHg or diastolic blood pressure (DBP) ≥ 90 mmHg, or a positive answer to “The doctor said you have high blood pressure.” Systolic and diastolic blood pressure values were assessed three to four times with a mercury sphygmomanometer using a conventional protocol. Three measurements were averaged to determine the SBP and DBP. Hypercholesterolemia is defined using total serum cholesterol: serum total cholesterol ≥ 200 mg/dL or “your doctor has said you have elevated cholesterol levels” or both. Diabetes was defined as the participant’s self-reported diagnosis or glycated hemoglobin (HbA1c) ≥ 6.5% or both. A further detailed description of examination protocol, quality control, and safety procedures is available on the NHANES website.

### Covariates

Baseline information on demographics and lifestyles was gathered utilizing a standardized questionnaire. Age was the age at the time screening was performed. The race was classified as non-Hispanic white and other racial groups (non-Hispanic black, non-Hispanic Asian, Mexican-American, other Hispanic groups, and other races). Marital status was divided into married and other (widowed, divorced, separated, never married, living with a partner). The ratio of family income to poverty means the ratio of family income to poverty guidelines. Smokers were defined as participants who had smoked at least 100 cigarettes during their lifetime. Drinking is defined as no drinking and more than one drink per drink. The Physical Activity Questionnaire’s activity type and intensity determine activity-specific MET values (46–49). Participants were divided into low and high physical activity categories before analysis (low physical activity was defined as 500 MET/week or less; high physical activity was defined as 500 MET/week or more) (50). Qualified researchers take anthropometric measurements like height, weight, arm circumference, and waist circumference and are taken by standard protocols. BMI was calculated as weight (kg) divided by standing height squared (m^2^). Serum samples were processed, stored under appropriate refrigeration (2-8°C), and shipped to the University of Minnesota Advanced Research Diagnostic Laboratory (ARDL) for analysis. Detailed specimen collection and processing instructions are discussed in the NHANES Laboratory Procedures Manual (LPM).

### Statistical analysis

NHANES has a complex, multistage, probability cluster design. We processed the data according to the tutorials provided by NHANES; this included weighting according to sample weights and multi-period combined weights and the underestimation of variance due to this design scheme adjustments. Multiple imputations were used to impute variables with missing values. Characteristics of the case and control groups were compared in each of the three outcomes, χ2 tests were used to compare categorical variables, and T-student tests to compare continuous variables.

**Figure 1:**
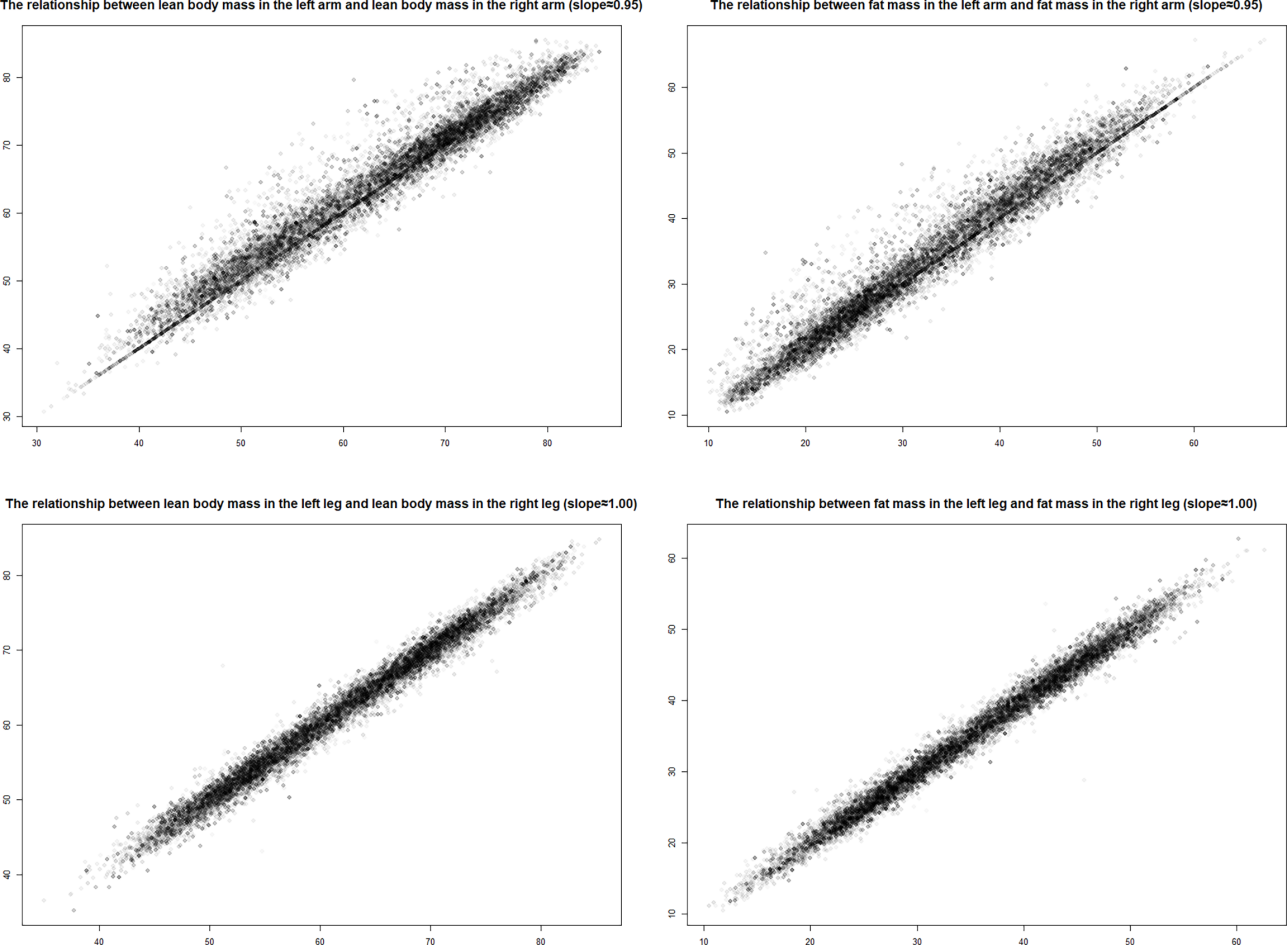
Lean mass and body fat percentage in left and right.

According to preliminary analysis, fat and lean body mass on the left and right are closely related (**Figure 1**), so LM% and FM% are represented by the average. The study expressed arm, leg, torso, and total LM and FM percent as quartiles and examined them as rank variables since body composition measures were not distributed normally. The first quartile was considered as a reference to explain any connections between body composition and MD, as reported by other studies on the NHANES population.

The correlations between segmental body composition quartiles for hypertension, hypercholesterolemia, and diabetes were estimated using binary logistic regression models. Odds ratios (OR) and 95% confidence intervals (95% CI) are provided. The model included body composition parameters separately to avoid over-tuning due to high correlations. The model was first adjusted for age and gender to form Model 1; on this basis, multi-factor adjustments were made to further adjust for the race, marital status, family income, smoking, drinking, physical activity, HbA1c, high-density lipoprotein-cholesterol (HDL-C), triglycerides, total cholesterol, SBP, DBP.

Further analyses were stratified by age and sex. The age subgroup analysis was divided into a middle-aged group (age >40) and a youth group (age ≤40) because the participants’ ages ranged from 20 to 59. Data were analyzed using R 4.1.0; all tests were two-sided, and P<0.05 was considered statistically significant.

## Results

### Participant characteristics

The characteristics of the participants are shown in **Table 1**. The case group had a higher BMI, larger arm and waist circumferences, and was more likely to be older, married, smokers, and less physically active. Compared to the non-hypercholesterolemia group, the hypercholesterolemia group had a higher percentage of men, non-Hispanic whites, and higher incomes. The diabetic group had fewer non-Hispanic people, fewer drinkers, and lower incomes than the non-diabetic group.

**Table 1:** Baseline characteristics in case and control groups: NHANES 2011-2018.

Characteristics	Hypertension (N=3569)	Non-Hypertension (N=8579)	P _value_	Hypercholesterolemia (N=5683)	Non-Hypercholesterolemia (N=6465)	P _value_	Diabetes (N=1212)	Non-Diabetes (N=10936)	P _value_
Age, y	45.91(0.21)	37.33(0.25)	<0.001	44.36(0.23)	35.42(0.24)	<0.001	48.02(0.31)	39.02(0.23)	<0.001
Female, %	46.82	50.94	<0.001	47.18	51.97	0.01	49.67	49.73	0.46
Non-Hispanic White, %	33.73	35.30	0.94	36.39	33.47	<0.001	24.50	35.98	<0.001
Married, %	50.35	47.59	<0.001	55.36	42.29	<0.001	56.52	47.50	<0.001
Smoker, %	47.46	36.52	<0.001	43.57	36.37	<0.001	43.81	39.28	<0.001
Drinker, %	68.93	71.31	0.099	70.33	70.86	0.735	61.39	71.63	<0.001
High physical activity, %	63.43	71.26	<0.001	65.69	71.83	<0.001	56.93	70.29	<0.001
Income	2.92(0.06)	2.94(0.05)	0.77	3.14(0.05)	2.74(0.05)	<0.001	2.72(0.08)	2.95(0.05)	<0.001
Arm circumference, cm	35.54(0.14)	32.92(0.10)	<0.001	34.18(0.12)	33.16(0.11)	<0.001	36.81(0.21)	33.38(0.09)	<0.001
Waist circumference, cm	106.78(0.47)	95.52(0.34)	<0.001	101.71(0.44)	95.83(0.40)	<0.001	113.32(0.75)	97.40(0.33)	<0.001
BMI, kg/m^2^	32.00(0.19)	28.05(0.14)	<0.001	29.98(0.19)	28.39(0.16)	0.05	34.42(0.33)	28.70(0.14)	<0.001
SBP, mmHg	128.55(0.65)	112.18(0.43)	<0.001	119.44(0.48)	114.27(0.52)	<0.001	122.74(1.42)	116.25(0.36)	<0.001
DBP, mmHg	77.03(0.52)	68.25(0.27)	<0.001	72.95(0.31)	68.61(0.34)	<0.001	72.67(0.90)	70.54(0.27)	<0.001
HDL-C, mmol/l	1.29(0.01)	1.37(0.01)	<0.001	1.36(0.01)	1.34(0.01)	<0.001	1.16(0.01)	1.36(0.01)	0.02
Triglyceride, mmol/l	2.01(0.04)	1.56(0.03)	<0.001	2.13(0.03)	1.27(0.02)	<0.001	2.51(0.10)	1.61(0.02)	0.35
Total cholesterol, mmol/l	5.08(0.03)	4.83(0.02)	<0.001	5.62(0.03)	4.24(0.01)	<0.001	4.95(0.05)	4.90(0.02)	<0.001
HbA1c, %	5.84(0.02)	5.41(0.01)	<0.001	5.71(0.02)	5.37(0.01)	<0.001	7.70(0.06)	5.35(0.01)	<0.001
Arm lean mass, %	61.39(0.20)	58.80(0.35)	<0.001	61.69(0.22)	59.56(0.25)	<0.001	61.07(0.18)	56.04(0.52)	<0.001
Arm fat mass, %	32.45(0.18)	34.01(0.23)	<0.001	32.21(0.20)	33.61(0.22)	<0.001	32.61(0.15)	36.07(0.38)	<0.001
Leg lean mass, %	61.45(0.17)	61.20(0.21)	0.36	61.49(0.17)	61.27(0.20)	0.24	61.39(0.15)	61.29(0.34)	0.44
Leg fat mass, %	35.21(0.18)	35.63(0.22)	0.15	35.18(0.18)	35.49(0.21)	0.37	35.31(0.15)	35.62(0.36)	0.81
Torso lean mass, %	67.21(0.18)	63.76(0.21)	<0.001	67.58(0.21)	64.81(0.19)	<0.001	66.65(0.16)	61.49(0.29)	<0.001
Torso fat mass, %	31.17(0.19)	34.80(0.22)	<0.001	30.75(0.22)	33.71(0.20)	<0.001	31.76(0.17)	37.10(0.29)	<0.001
Total lean mass, %	64.53(0.15)	63.10(0.18)	<0.001	64.73(0.16)	63.48(0.16)	<0.001	64.30(0.13)	62.19(0.28)	<0.001
Total fat mass, %	32.46(0.16)	34.22(0.19)	<0.001	32.23(0.17)	33.72(0.17)	<0.001	32.75(0.14)	35.32(0.28)	<0.001

Data are mean (SE) or percentage. P value was estimated using χ^2^ for proportions, T test for means. NHANES, National Health and Nutrition Examination Survey; Income: A ratio of family income to poverty guidelines; BMI, body mass index (calculated as weight in kilograms divided by height in meters squared); HDL-C, high density lipoprotein -Cholesterol; HbA1c, glycated hemoglobin A1c. All estimates accounted for complex survey designs, and all percentages were weighted.

### Association of body composition parameters with metabolic disease

**Table 2** illustrates the relationship between body composition characteristics and MD. After adjustment of age and gender, participants in the highest quartile of the arm LM% [hypertension (OR=0.83, 95%CI: 0.79, 0.87), hypercholesteremia (OR=0.86, 95%CI: 0.82, 0.91), diabetes (OR=0.89, 95%CI: 0.87, 0.91)], torso LM% [hypertension (OR=0.80, 95%CI: 0.77, 0.83), hypercholesteremia (OR=0.84, 95%CI: 0.80, 0.88), diabetes (OR=0.88, 95%CI: 0.86, 0.89)], and total LM%[hypertension (OR=0.86, 95%CI: 0.82, 0.89), hypercholesteremia (OR=0.89, 95%CI: 0.85, 0.93), diabetes (OR=0.93, 95%CI: 0.91, 0.95)] had a lower risk of metabolic disease.

**Table 2:** Associations of body composition parameters with Hypertension, Hypercholesteremia, Diabetes in NHANES 2011-2018.

Body composition parameters		Hypertension				Hypercholesteremia				Diabetes			
	N	model 1	P _trend_	model 2	P _trend_	model 1	P _trend_	model 2	P _trend_	model 1	P _trend_	model 2	P _trend_
**Arm LM%**													
Q1(16.05-51.96)	3065	reference	<0.001	reference	<0.001	reference	<0.001	reference	<0.001	reference	<0.001	reference	<0.001
Q2(51.96-62.35)	3065	0.93(0.91,0.96)		0.96(0.93,0.98)		0.96(0.93,0.99)		0.97(0.95,1.00)		0.97(0.95,0.98)		1.00(0.98,1.01)	
Q3(62.35-70.03)	2890	0.92(0.89,0.95)		0.96(0.93,0.99)		0.95(0.91,0.99)		0.97(0.95,1.00)		0.93(0.91,0.95)		0.98(0.96,0.99)	
Q4(70.03-84.39)	3128	0.83(0.79,0.87)		0.88(0.84,0.92)		0.86(0.82,0.91)		0.93(0.89,0.96)		0.89(0.87,0.91)		0.96(0.95,0.98)	
**Arm FM%**													
Q1(5.80-23.75)	3108	reference	<0.001	reference	<0.001	reference	<0.001	reference	0.07	reference	<0.001	reference	0.014
Q2(23.75-31.30)	2894	1.09(1.05,1.12)		1.07(1.04,1.09)		1.10(1.07,1.14)		1.03(1.00,1.06)		1.03(1.01,1.04)		1.00(0.99,1.02)	
Q3(31.30-42.05)	2970	1.09(1.06,1.13)		1.06(1.03,1.09)		1.10(1.06,1.15)		1.03(1.00,1.06)		1.05(1.03,1.08)		1.02(1.00,1.03)	
Q4(42.05-67.20)	3176	1.19(1.15,1.23)		1.11(1.07,1.15)		1.17(1.11,1.23)		1.05(1.01,1.09)		1.10(1.08,1.13)		1.03(1.01,1.05)	
**Leg LM%**													
Q1(36.44-53.56)	2996	reference	<0.001	reference	<0.001	reference	0.01	reference	0.36	reference	0.84	reference	0.42
Q2(53.56-61.52)	3066	0.99(0.96,1.02)		1.00(0.97,1.03)		0.99(0.96,1.03)		1.00(0.97,1.03)		1.02(1.00,1.04)		1.02(1.00,1.03)	
Q3(61.52-69.18)	2964	0.97(0.94,1.01)		0.99(0.95,1.02)		1.01(0.97,1.05)		1.01(0.98,1.05)		1.01(0.99,1.04)		1.01(0.99,1.03)	
Q4(69.18-85.04)	3122	0.90(0.86,0.94)		0.93(0.89,0.97)		0.97(0.92,1.02)		0.99(0.95,1.04)		1.00(0.97,1.02)		1.01(0.99,1.03)	
**Leg FM%**													
Q1(10.70-27.20)	3121	reference	<0.001	reference	<0.001	reference	0.004	reference	0.27	reference	0.37	reference	0.54
Q2(27.20-35.15)	2959	1.08(1.04,1.12)		1.06(1.03,1.09)		1.04(1.01,1.08)		1.02(0.99,1.05)		1.02(1.00,1.03)		1.00(0.99,1.02)	
Q3(35.15-43.50)	3070	1.10(1.06,1.15)		1.08(1.04,1.12)		1.04(1.00,1.08)		1.01(0.97,1.05)		1.02(1.00,1.04)		1.00(0.99,1.02)	
Q4(43.50-61.85)	2998	1.12(1.07,1.17)		1.08(1.03,1.13)		1.05(1.00,1.10)		1.01(0.97,1.06)		1.01(0.99,1.04)		0.99(0.97,1.01)	
**Torso LM%**													
Q1(41.18-59.74)	3183	reference	<0.001	reference	<0.001	reference	<0.001	reference	<0.001	reference	<0.001	reference	<0.001
Q2(59.74-66.27)	3070	0.91(0.88,0.94)		0.95(0.92,0.97)		0.98(0.95,1.01)		0.99(0.97,1.02)		0.93(0.91,0.95)		0.98(0.96,1.00)	
Q3(66.27-72.37)	2933	0.84(0.82,0.87)		0.90(0.87,0.93)		0.94(0.91,0.98)		0.98(0.95,1.01)		0.90(0.88,0.91)		0.97(0.95,0.99)	
Q4(72.37-92.56)	2962	0.80(0.77,0.83)		0.86(0.83,0.89)		0.84(0.80,0.88)		0.94(0.91,0.98)		0.88(0.86,0.89)		0.96(0.95,0.98)	
**Torso FM%**													
Q1(10.10-25.90)	2978	reference	<0.001	reference	<0.001	reference	<0.001	reference	<0.001	reference	<0.001	reference	<0.001
Q2(25.90-32.20)	2933	1.07(1.04,1.10)		1.06(1.03,1.09)		1.13(1.09,1.17)		1.04(1.01,1.07)		1.02(1.01,1.03)		1.00(0.99,1.01)	
Q3(32.20-38.80)	3062	1.14(1.11,1.17)		1.10(1.08,1.13)		1.17(1.12,1.21)		1.05(1.02,1.09)		1.06(1.04,1.07)		1.02(1.01,1.03)	
Q4(38.80-58.00)	3175	1.26(1.22,1.31)		1.18(1.14,1.22)		1.20(1.15,1.26)		1.06(1.03,1.10)		1.14(1.12,1.16)		1.03(1.02,1.05)	
**Total LM%**													
Q1(42.99-57.82)	3149	reference	<0.001	reference	<0.001	reference	<0.001	reference	0.17	reference	<0.001	reference	0.1
Q2(57.82-64.51)	3049	0.94(0.91,0.97)		0.97(0.95,1.00)		0.96(0.93,1.00)		1.00(0.97,1.03)		0.97(0.96,0.99)		1.00(0.98,1.01)	
Q3(64.51-70.15)	2920	0.94(0.91,0.97)		0.98(0.95,1.01)		0.97(0.93,1.01)		1.01(0.98,1.04)		0.97(0.95,0.99)		1.00(0.99,1.02)	
Q4(70.15-84.37)	3030	0.86(0.82,0.89)		0.91(0.88,0.94)		0.89(0.85,0.93)		0.98(0.94,1.02)		0.93(0.91,0.95)		0.99(0.97,1.01)	
**Total FM%**													
Q1(11.70-26.90)	3090	reference	<0.001	reference	<0.001	reference	<0.001	reference	0.08	reference	<0.001	reference	0.04
Q2(26.90-32.60)	2889	1.10(1.07,1.13)		1.08(1.05,1.11)		1.10(1.06,1.14)		1.03(1.00,1.07)		1.04(1.02,1.06)		1.02(1.00,1.03)	
Q3(32.60-39.40)	3040	1.12(1.09,1.16)		1.09(1.06,1.12)		1.10(1.05,1.14)		1.03(0.99,1.06)		1.05(1.03,1.07)		1.02(1.01,1.03)	
Q4(39.40-56.10)	3129	1.19(1.15,1.24)		1.12(1.08,1.16)		1.14(1.09,1.19)		1.03(0.99,1.07)		1.08(1.06,1.10)		1.01(1.00,1.03)	

LM%, lean mass percentage; FM%; fat mass percentage.

Model 1: Adjusted for age and gender.

Model 2: Adjusted for age, gender, race, marital status, the ratio of family income to poverty, smoking, alcohol consumption, physical activity, HbA1c, HDL-C, triglycerides, SBP, DBP, total cholesterol.

An opponent association was found for the arm FM% [hypertension (OR=1.19, 95%CI: 1.15, 1.23), hypercholesteremia (OR=1.17, 95%CI: 1.11, 1.23), diabetes (OR=1.10, 95%CI: 1.08, 1.13)], torso FM%[hypertension (OR=1.26, 95%CI: 1.22, 1.31), hypercholesteremia (OR=1.20, 95%CI: 1.15, 1.26), diabetes (OR=1.14, 95%CI: 1.12, 1.16)], and total FM% [hypertension (OR=1.19, 95%CI: 1.15, 1.24), hypercholesteremia (OR=1.14, 95%CI: 1.09, 1.19), diabetes (OR=1.08, 95%CI: 1.06, 1.10)].

Except for the total LM% and total FM% in hypercholesteremia and diabetes, this association is constant even after accounting for several factors. A similar relationship was not generally found in leg body composition, only in the relationship between leg fat mass percentage and hypertension.

### The relationship of segmental body composition on metabolic disease across age and gender

**Figure 2:**
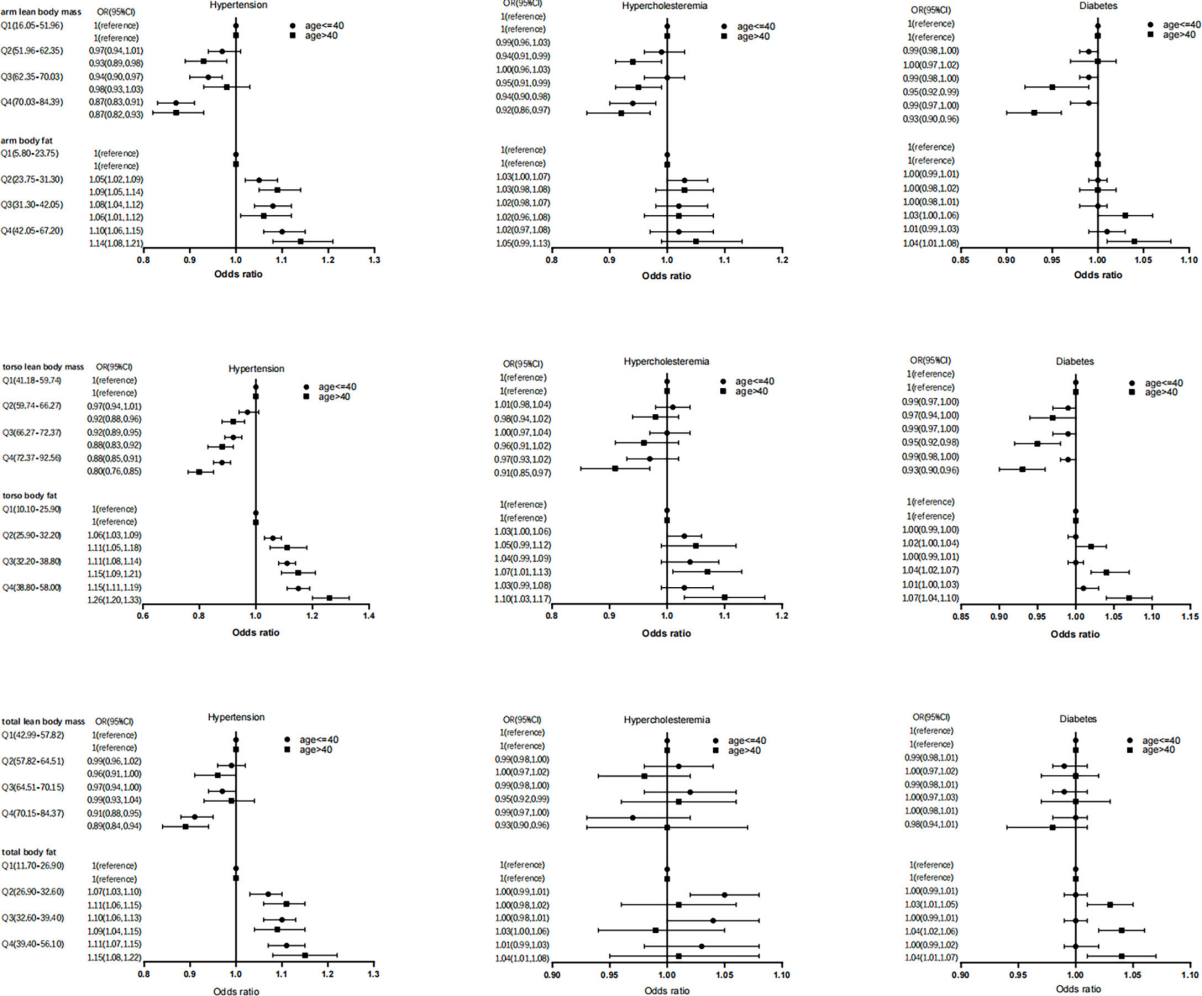
Association of segmental body composition with metabolic disease, at different ages.

Based on Model 2, a subgroup analysis was conducted, and the three outcomes yielded various findings. We discovered no discernible interaction between age and body composition characteristics for determining the risk of hypertension (**Figure 2**). The protective effect of lean body mass is observed to be larger in middle-aged individuals than in young adults in the subgroup analysis of hypercholesterolemia, particularly in arm LM% [age>40 (OR=0.92, 95%CI: 0.86, 0.97) vs. age ≤ 40 (OR=0.94, 95%CI: 0.90, 0.98)] and torso LM%[age>40 (OR=0.91, 95%CI: 0.85, 0.97) vs. age ≤ 40 (OR=0.97, 95%CI: 0.93, 1.02)] (**Figure 2**). In the subgroup analysis of diabetes, we can be found same relationship in the arm LM% [age>40 (OR=0.93, 95%CI: 0.90, 0.96) vs. age ≤ 40 (OR=0.99, 95%CI: 0.97, 1.00)] and torso LM% [age>40 (OR=0.93, 95%CI: 0.90, 0.96) vs. age ≤ 40 (OR=0.99, 95%CI: 0.98, 1.00)] (**Figure 2**). When assessing the risk of diabetes, the risk effect of segmental FM% gain is considerably bigger in middle-aged individuals than in young adults [arm body fat (OR=1.04, 95%CI: 1.01, 1.08), torso body fat (OR=1.07, 95%CI: 1.04, 1.10), total body fat (OR=1.04, 95%CI: 1.01, 1.07)] (**Figure 2**).

**Figure 3:**
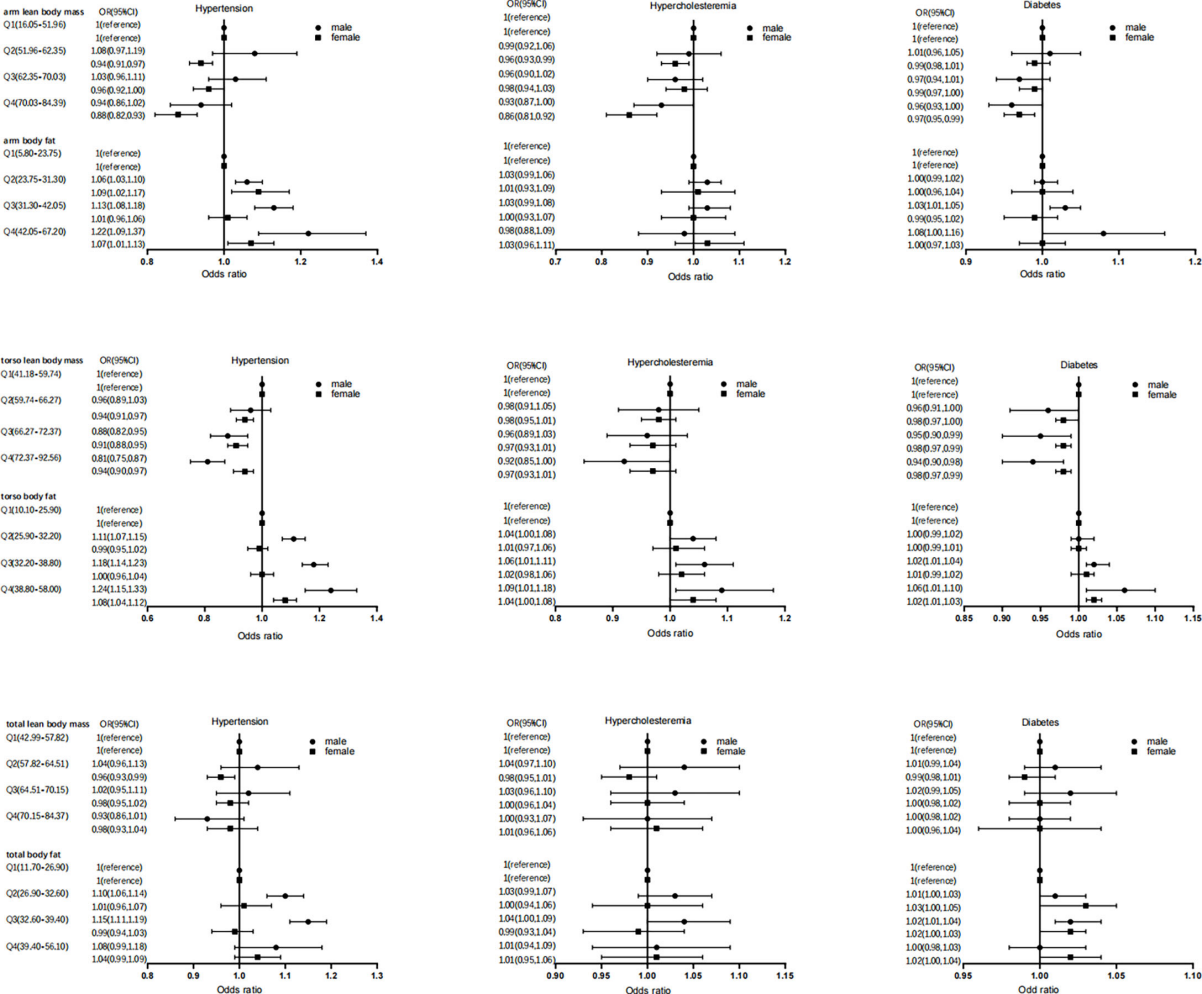
Association of segmental body composition with metabolic disease, at different gender.

Meanwhile, gender differences exist in the impact of segmental body composition on metabolic disease (**Figure 3**). Increased LM% had a stronger protective effect on metabolic disease in women, particularly in the arm [hypertension (OR=0.88, 95%CI: 0.82, 0.93), hypercholesteremia (OR=0.86, 95%CI: 0.81, 0.92), diabetes (OR=0.97, 95%CI: 0.85, 0.99)] (**Figure 3**). Conversely, increased FM% was associated with a higher risk of metabolic disease in men, particularly in torso FM% [hypertension (OR=1.24, 95%CI: 1.15, 1.33), hypercholesteremia (OR=1.09, 95%CI: 1.01, 1.18), diabetes (OR=1.06, 95%CI: 1.01, 1.10)] (**Figure 3**).

## Discussion

According to our study’s findings on segmental body composition, the percentage of lean body mass and body fat in the arm and torso were strongly associated with metabolic disease. The association persisted, and the trend remained statistically significant after potential confounders were considered. On this basis, we also found that when assessing metabolic disease risk, body fat has a more substantial effect in men and lean body mass has a more significant impact in women. For middle-aged adults (age > 40 years), upper limb lean body mass and torso body fat had more significant effects on hypercholesterolemia and diabetes than young adults (age ≤ 40 years). Because of this, our findings imply that segmental body composition characteristics are essential to include when evaluating metabolic risk.

To our knowledge, this is the first study to examine the relationship between segmental body composition and metabolic disease. According to a survey conducted on black Africans, body composition is not the leading cause of high blood pressure (35). Another study conducted among South Asians found no strong evidence that body composition could explain type 2 diabetes risk differences (34). Contrary to our findings, which may be caused by different methods of assessing body composition and different ethnic groups in the study population. In a study from the Korea National Health and Nutrition Examination Survey (51), it was discovered that among non-obese and obese individuals in the lowest tertile of the leg fat ratio to total fat, there was a decreased prevalence of hypertension, diabetes, and metabolic syndrome. This differs from our findings because the participants in our study had a higher BMI and were from different ethnicities. Furthermore, cohort studies (52) have shown that body fat distribution in women has shifted from the lower to the upper body in recent years, which may also be responsible for the disparity. According to a study from a Chinese population, the total skeletal muscle index and body fat % were substantially linked to high OR in pre-hypertension and hypertension, and arm lean body mass was more closely correlated with systolic and diastolic blood pressure than leg lean body mass (53). In a Korean study, men’s torso fat mass percentage was strongly correlated with hypercholesterolemia and was closely associated with hypertension (54). Similar to our findings, we analyzed multiple outcomes and segmental body compositions simultaneously.

The mid-upper arm circumference (MUAC) is regarded as a straightforward and reliable criterion for assessing obesity (55) and screening fat distribution (56) previously. However, these studies were conducted on children. Recent research has shown that MUAC can be used to detect central obesity and insulin resistance (57) and diagnose sarcopenia (58). Shi et al. (59)showed that MUAC was significantly associated with metabolic syndrome in middle-aged and older people. According to research, upper arm obesity may be a sign of central obesity, systemic obesity, or sarcopenia (60). Most of these studies were conducted in Asia, and we do not know if these conclusions hold in Americans. Although it makes sense to use MUAC to evaluate metabolic disease risk, additional research is required to comprehend this phenomenon fully. Based on MUAC, our study further proved the relationship between upper arm body composition and metabolic disease.

This study reveals that biologically based hypertension, hypercholesterolemia, and diabetes are related to the body composition of the upper arm and torso. In addition to reducing strength, muscle loss may also disrupt normal metabolism. First, the Skeletal muscle is the leading site of glucose utilization. A decrease in muscle mass is associated with a lower basal metabolic rate. It exacerbates insulin resistance (61), an established risk factor for hypertension (62), and affects the development of diabetes. Loss of muscle mass may enhance inflammation and oxidative pathways (62), associated with metabolic disease risk (63, 64). The second, loss of muscle mass, is associated with increased arterial stiffness (65), which may mediate hypercholesterolemia and hypertension (62, 66). In recent years, studies have shown that skeletal muscle functions as an endocrine organ that can produce and secrete hundreds of muscle factors associated with adverse clinical outcomes in patients with cardiovascular disease (67). Conversely, abdominal obesity might induce sarcopenia *via* the activation of proinflammatory cytokines, such as interleukin-6 and tumor necrosis factor-a. Narasimhulu et al. reported that increased hyperglycaemia and inflammation are associated with cellular pyroptosis, leading to significant loss of muscle cells and adverse remodelling (68).

This study has important clinical implications. We noted that the arm and torso body composition were strongly associated with metabolic disease. This finding provided indirect evidence that arm and torso body composition may better reflect whether there is a metabolic disorder than other segmental body composition parameters. Increasing muscle mass, particularly in the muscles of the upper limbs, had a more significant protective impact against metabolic diseases in women. For men, maintaining body fat in the low range is more conducive to reducing the risk of metabolic diseases. In the clinical analysis of body composition, more attention should be paid to the distribution of fat and lean body mass in the arm and torso. Targeting this link between segmental body composition and metabolic disease can be countered by protein supplementation (69) and increased resistance exercise (70). Sex hormones are known to affect muscle mass (71, 72). In earlier animal studies, male rats were also more susceptible to the harmful effects of diabetes on body composition than female rats (73). Estrogen is an antioxidant and sarcolemmal stabilizer that appears crucial for muscle protein turnover, benefits skeletal muscle contractile abilities, and guards against muscle deterioration (74). Testosterone is involved in energy balance, glucose metabolism, insulin sensitivity, and lipid metabolism. Low testosterone levels are associated with increased fat mass (especially central obesity) and decreased lean mass in men (75). Reduced sex hormone secretion with age (76) may also explain the effect of body composition on the onset of metabolic diseases in middle-aged people.

The advantage of this study is that the sample size is large. We strictly follow the variance estimation and weighted processing scheme provided by NHANES, and we use the latest DXA data and be sure to be contemporaneous. However, we also acknowledge that there are some limitations to the study. First, the type of study is cross-sectional, which is bound to limit the determination of causality. Because of this, there may be a potential reverse causality, in which chronic metabolic abnormalities lead to segmental muscle loss and fat accumulation. Prospective cohort studies are needed in future studies to assess the order of these associations. Second, after menopause, estrogen levels decrease muscle mass decreases, and fat mass increases (77). However, in this study, the age was limited to 59 years old, so the number of postmenopausal women was negligible. Third, participants with invalid DXA data were excluded, partly because of excess body weight, although this part of the data was not significant. Finally, despite the exclusion of minors, the participants were relatively young, depending on the conventional demographic age structure. It may have prevented our results from generalized to other groups, such as the elderly (age>60).

## Conclusions

In conclusion, we report the association between segmental body composition and metabolic disease. In the upper limbs and torso, increased lean body mass is a protective factor for metabolic disease, and a higher fat percentage is a risk factor for metabolic disease. This relationship varies by sex and age. Our results imply that, in addition to overall body fat and lean mass percentage, we should consider body composition in upper limbs and torso segments when assessing metabolic disease risk. However, additional cohort studies are required to confirm these findings.

## Data availability statement

Publicly available datasets were analyzed in this study. This data can be found here: https://wwwn.cdc.gov/nchs/nhanes/Default.aspx.

## Ethics statement

The studies involving human participants were reviewed and approved by NCHS Research Ethics Review Board (ERB) Approval. The patients/participants provided their written informed consent to participate in this study.

## Author contributions

LF was responsible for funding acquisition. QQ and KS contributed to study design. YR, ZL, LF, YW, DZ, SS and HW carried out the clinical assessments. QQ and KS were responsible for data curation. QQ and KS analyzed the data. QQ wrote the manuscript, which was critically reviewed by all other authors. All authors contributed to the article and approved the submitted version.

## Funding

On this basis, the work was supported by the key research and development plan of Shandong Province (2016GSF201007).

## Conflict of interest

The authors declare that the research was conducted in the absence of any commercial or financial relationships that could be construed as a potential conflict of interest.

## Publisher’s note

All claims expressed in this article are solely those of the authors and do not necessarily represent those of their affiliated organizations, or those of the publisher, the editors and the reviewers. Any product that may be evaluated in this article, or claim that may be made by its manufacturer, is not guaranteed or endorsed by the publisher.
